# Efficacy of Ezetimibe/Simvastatin (10/10 mg) versus High Dose Statin in Dyslipidemia Patients: A Meta-Analysis of Randomized Controlled Trials

**Published:** 2019-08

**Authors:** Gaoming YANG, Dengfeng HAN, Jianhua MA, Xiaoning ZHANG

**Affiliations:** 1. Department of Neurology, First Affiliated Hospital of Xinjiang Medical University, Urumqi, 830054, China; 2. Department of Neurology, Second Affiliated Hospital of Chengdu Medical College (China National Nuclear Corporation 416 Hospital), Chengdu, 610000, China; 3. Department of Neurology, Traditional Chinese Medicine Hospital Affiliated to Xinjiang Medical University, Urumqi, 830054, China

**Keywords:** Ezetimibe, Statin, Cholesterol, Inflammation, Meta-analysis

## Abstract

**Background:**

The monotherapies of statin and ezetimibe had not successfully achieved their objectives in the management of lipid levels of dyslipidemia patients. We aimed to compare the effects of combined low-dose simvastatin and ezetimibe versus high-dose statin on the lipid-lowering treatment of dyslipidemia patients.

**Methods:**

We searched five databases published before May 2018, namely PubMed, EMBASE, Cochrane, Web of Science, and Clinicaltrials.gov. Completely published randomized controlled trials (RCTs) comparing the effect of high-dose statin (S) with ezetimibe/simvastatin (10/10 mg; E/S) on the management of dyslipidemia patients were included.

**Results:**

A total of ten RCTs met the inclusion criteria, including 1,624 patients (E/S:691, S:933). Six outcomes underwent pooled analysis, including weighted mean difference (WMD) from baseline in total cholesterol (TC), low-density lipoprotein cholesterol (LDL-C), high-density lipoprotein cholesterol (HDL-C), high sensitivity C-reactive protein (hs-CRP), triglyceride (TG), and non-high-density lipoprotein cholesterol (non-HDL-C). No significant gap was found between high-dose statin and ezetimibe/simvastatin (10/10 mg) in LDL-C (−1.55; 95% confidence interval [CI]: −4.42∼1.31, *P*=0 .29), HDL-C (1.05; 95%CI: −0.21∼2.3, P=0 .1), TG (4.03; 95%CI: −4.53∼12.58, *P*=0.36), and hs-CRP (0.14; 95%CI: −0.50∼0.78, *P*=0.67). However, there was significant difference found between the two lipid-lowering treatments in TC (−0.45; 95%CI: −9.07∼−0.83, P=0.02) and non-HDL-C (−4.97; 95%CI −8.46∼−1.49, *P*=0.005).

**Conclusion:**

Ezetimibe co-administered with simvastatin (10 mg) and high-dose statin monotherapy may show similar effects in reducing LDL-C, TG, and hs-CRP levels and in increasing HDL-C levels. However, the results suggest that there was greater TC and non-HDL-C lowering through high-dose statin monotherapy as compared with ezetimibe/simvastatin co-administration.

## Introduction

Ischemic stroke, coronary heart disease (CHD), and peripheral arterial disease (PAD) were called atherosclerotic cardiovascular disease (ASCVD) (1). The data suggested the age-standardized death rate attributable to all ASCVD in the US population was 223.9 per 100,000 in 2013 (2). Meanwhile, stroke is the first and the third leading cause of death in rural and urban areas in China (3). In 2005, ASCVD is the most common cause of death in the UK, in which 49% of deaths are due to CHD and about 28% are due to stroke (4). Dyslipidemia, particularly, low-density lipoprotein cholesterol (LDL-C), is a major contributor to the formation and development of atherosclerotic plaques (5). Inflammation is a key participant in atherosclerosis formation by intervening or modulating systemic and local inflammatory responses. Evidences from experimental model studies support the viewpoint that inflammation is a driver of atherosclerosis. High sensitivity C-reactive protein (hs-CRP) is an indicator of subclinical inflammation. Furthermore, all these factors can lead to the development of ASCVD.

The management of dyslipidemia or hypercholesterolemia is constantly evolving. The treatment aims to prevent or reduce the risk and complications of ASCVD (6). There are two primary ways to manage hypercholesterolemia: through lifestyle changes, such as proper diet, exercise, and weight management, and through medication (7). The 3-hydroxy-3-methylgutaryl coenzyme A reductase inhibitors or statins are recommended as the first-line drug to treat hypercholesterolemia (1, 8, 9). Aside from its lipid-lowering efficacy, statins can also reduce infarct volume and increase neurological function through its anti-inflammatory, antithrombotic, anti-oxidant, anti-apoptotic, and neuroprotective properties (1). Statin is a well-established and effective medication to lower lipid levels. It can increase high-density lipoprotein cholesterol (HDL-C) levels from 2.3 to 7.9%, and it can decrease LDL-C, non-HDL-C, triglyceride (TG) levels within ranges of 27–55%, 25–50%, 9–25%, respectively (10, 11). Although statin is proven to be beneficial in the management of hypercholesterolemia, some patients given the highest doses of statin still fail to achieve their LDL-C target levels (10). However, statin has numerous adverse effects (AEs) such as myalgia, myopathy, rhabdomyolysis, pathoglycemia, hepatotoxicity, etc (5, 10, 12, 13). High-intensity statin (atorvastatin 40–80 mg, rosuvastatin 20–40 mg, and simvastatin 80 mg) was able to reduce LDL-C about > 50% from the untreated baseline (14). However, there is a close relationship between high dosage of statins and increased risk of AEs (10).

Ezetimibe is an epoch-making inhibitor of intestinal cholesterol absorption (15). The inhibition of absorption of bile acid-derived reabsorbed and food-derived cholesterols is associated with the mechanism of ezetimibe. It could significantly reduce levels of postprandial TG, reduce LDL-C rate by 23%, and increase the effect of statin to reduce serum TG (16, 17). Ezetimibe and statin have some similar effects, and they are both involved in ameliorating oxidative stress, insulin resistance, and athero-sclerotic and inflammatory markers (9). Over the past years, the Randomized Controlled Trials (RCTs) on ezetimibe plus statin and/or statin monotherapy constantly evolved, and example of such studies are Ezetimibe and Simvastatin in Hypercholesterolemia Enhances Atherosclerosis Regression (ENHANCE), Simvastatin and Ezetimibe in Aortic Stenosis (SEAS), Study of Heart and Renal Protection (SHARP), and Improved Reduction of Outcoms: Vytorin Efficacy International Trial (IMPROVE-IT) (18–21).

Some studies and guides recommended ezetimibe in combination with a statin to reduce the risk of ASCVD events for at-risk patients (22, 23). However, it is still controversial whether there is a difference in the effects of combined, low-dose simvastatin and ezetimibe as compared with high-dose statin on lipid-lowering treatment.

Hence, we performed the meta-analysis to compare the effect of the combination of low-dose simvastatin and ezetimibe (defined as daily dose of ezetimibe/simvastatin 10/10 mg) with high-dose statin (defined as a daily dose of atorvastatin 40–80 mg, rosuvastatin 20–40 mg, and simvastatin 80 mg) on lipid-lowering treatment in dyslipidemia patients.

## Methods

Our meta-analysis was conducted according to the preferred reporting items for systematic reviews and meta-analyses (PRISMA) Statement (24). However, the data based on published studies, there are no ethical issues in this systematic review and meta-analysis.

### Data Searching

This systematic review and meta-analysis can be found in four common databases and one web-site: Clinicaltrials.gov . We searched Medline by PubMed, Embase, Cochrane Library databases, Web of Science, and Clinicaltrials.gov for completed or ongoing trials with the keywords “ezetimibe”, “simvastatin”, “atorvastatin”, “rosuvastatin”, and “statin”. The boolean logic and wildcard and truncation symbols were used in bibliographic retrieval. The final search was completed on May 2018. Through manual search, the references of the original manuscripts, review, and meta-analysis were useful in bibliographic retrieval. The search strategy for Medline by Pubmed is shown in detail in the electronic supplementary material (ESM 2).

### Study Selection

Two reviewers (G.M.Y. and D.F.H) searched and filtered the related articles, and disagreement was resolved by conformable discussion. The third reviewer (X.N.Z.) contributed to solve the discrepancies through discussion with the two previous reviewers.

Inclusion criteria: 1) RCTs; 2) comparison of low-dose simvastatin plus ezetimibe (defined as a daily dose of ezetimibe/simvastatin 10/10 mg) and high-dose statin (defined as a daily dose of atorvastatin 40–80 mg, rosuvastatin 20–40 mg, and simvastatin 80 mg); 3) assessment of the therapeutic effect of changes in LDL-C levels in hypercholesterolemia; and 4) reporting data (means and standard deviations [SD]) regarding pre-intervention and post-intervention LDL-C or change from baseline LDLC. Exclusion criteria: 1) non-human studies, 2) incomplete papers, 3) reviews, 4) observational studies, 5) post-hoc studies, 6) pooled analysis, 7) subgroup analysis, 8) letters, 9) conference summaries/ papers, 10) non-English papers.

### Data Extraction

Two reviewers (G.M.Y. and D.F.H) achieved data extraction from complete papers that passed the inclusion criteria, and disagreement was resolved through conformable discussion. The third reviewer (X.N.Z.) contributed to solve the discrepancies through discussion with the two previous reviewers. The following data were extracted: 1) the primary information: author, publication time, and study design; 2) profile of participant: headcount, gender, age, body mass index (BMI), diabetes or impaired glucose tolerance (IGT), hypertension, CHD, PAD, stroke, smoking habits; 3) intervention: type of drug, dosage, and duration of therapy; 4) outcome: pre-/post-intervention or change from the baseline values of LDL-C, HDL-C, total cholesterol (TC), TG, non-HDL-C, and hs-CRP.

### Risk of Bias Assessment

A systematic quality assessment was performed using the Cochrane Collaboration Risk of Bias Tool in the included RCTs. The study quality was independently evaluated by two reviewers (G.M.Y. and D.F.H), and the differences were settled through mutual discussion. The third reviewer (X.N.Z.) contributed to solve the discrepancies through discussion with the two previous reviewers. The risk of bias assessment included: 1) random sequence generation (selection bias); 2) allocation concealment (selection bias); 3) blinding of participants and personnel (performance bias); 4) blinding of outcome assessment (detection bias); 5) incomplete outcome data (attrition bias); 6) selective reporting (reporting bias); and 7) other biases. According to Cochrane Collaboration Risk of Bias Tool, a judgment of ‘yes’ revealed low risk of bias, whereas ‘no’ revealed high risk of bias, and ‘unclear’ revealed an unclear or unknown risk of bias (9, 25).

### Statistical analysis

The pre- and post-interventions or change from the baseline of LDL-C acted as the primary outcome, and the pre- and post-interventions or change from the baseline of HDL-C, TC, TG, non-HDL-C, and hs-CRP acted as the secondary outcome. The non-HDL-C was calculated as TC minus HDL-C, and this was assessed as mean or median± SD. The data extraction and count were applied to the change (mean or median±SD) in response to pre-/post-therapy. However, we failed to use SD in some (26–29). Thus, we utilized SD of baseline instead of SD of change.

RevMan 5.3 (Cochrane Collaboration, Oxford, UK) was used for the statistical analysis of the paper. The weighted mean difference (WMD) and its 95% confidence intervals (CIs) played an important role in the pooled effect. Heterogeneity was assessed using the Cochrane’s Q test. If the p value is greater than 0.10, low heterogeneity was considered. In addition, the I^2^ statistic was applied to assess heterogeneity as well. We considered levels of heterogeneity as follows: 1) I^2^ < 20%: low heterogeneity; 2) I^2^: 20–50%: evident heterogeneity; 3) I^2^: 50–75%: material heterogeneity; and 4) I^2^ > 75%: considerable heterogeneity. Low heterogeneity or I^2^ < 20% was determined using the mean of fixed effect model as calculated by RevMan 5.3. Other heterogeneities were determined using a random effect model.

We performed data merging based on heterogeneity levels. If the heterogeneity was not low, sensitive analysis or subgroup analysis was then applied. Publication bias was assessed through visual inspection of the funnel plots.

## Results

### Study Searching

**Fig. 1:**
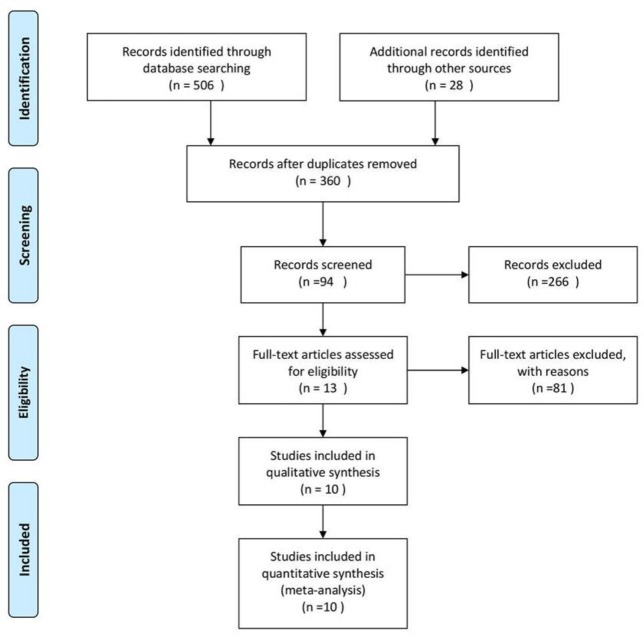
Process of literature searching

We accepted a total of ten studies included in 13 literatures through database and manual searching (Fig. 1). In those literatures, three studies have two reduplicative literatures. Finally, we included ten studies. A total of 691 patients in the ezetimibe/simvastatin group and a total of 933 patients in the high-dose statin group were included in this systematic review and meta-analysis. These studies were conducted within 4 to 14 weeks and included four parallel, three factorial, and three crossover studies.

### Study characteristics

The ten studies included in our meta-analysis (26–38) (Table 1). Among them, some had two reduplicative literatures including (31, 32), (27, 37), and (29, 38). The mean±SD or median±SD and change from baseline were widely used to the field of numerical variable. The sample sizes of the studies included 10 to 232 participants. The reported mean age of the participants in each study ranged from 41 to 74 years old. Data conversion was also a key procedure in the statistical analysis. However, the SD of change from baseline of some studies were not obtained; thus, SD in baseline was obtained instead (26, 27, 29).

**Table 1:** Summary details for included studies

***Studies***	***Participants***	***Intervention***	***Duration time, week***	***Sample size***	***Age, Year***	***Male, %***	***Race, %***
Araujo 2010 (30)	Hypercholesterolemia	E/S	4	11	NA	NA	NA
		10/10					
		S 80		12			
Settergren 2009 (31, 32)	Diabetes or IGT, stable CAD	E/S	6	15	74 (66–77)^a^	60	NA
		10/10					
		S 80		17	70 (67–74)^a^	76.47	
Olijhoek 2008 (33)	Metabolic syndrome	E/S	6	19	54±7	100	NA
		10/10					
		S 80		19			
Carcia 2016 (34)	Excess weight	E/S	8	16	48.0±8.1	0	NA
		10/10					
		S 80		16	41.0±8.6		
Goldberg 2004 (26)	Hypercholesterolemia	E/S	12	87	NA	48	White 83%, Black 3%, Hispanic 9%, Others 5%
		10/10					
		S 80		87		49	White 79%, Black 4%, Hispanic 10%, Others 7%
Rudofsky 2012 (35)	Diabetes	E/S	8	11	65±9	45.45	NA
		10/10					
		S 80		10	56±10	40	
Westerink 2013 (36)	Metabolic syndrome, abdominally obese patients	E/S	6	90	57±9	59	NA
		10/10					
		S 80		91			
Ose 2007 (27, 37)	Hypercholesterolemia	E/S	14	151	56 (22–80)^b^	46	White 88%, Black 3%, Hispanic 1%, Others 8%
		10/10					
		S 80		156	55 (22–83)^b^	48	White 88%, Black 3%, Hispanic 3%, Others 7%
Ballantyne 2005 (28)	Hypercholesterolemia	E/S	6	230	E/S, 59.0±10.6	E/S, 52.2	E/S:White 86.3%, Black 7.6%, Hispanic 4.4%, Others 1.7%
		10/10					
		A 40		232	A, 58.5±10.2	A, 52.4	A:White 86.0%, Black 7.5%, Hispanic 4.7%, Others 1.8%
		A 80		230			
Davidson 2002 (29, 38)	Hypercholesterolemia	E/S	12	61	57.6 (27–83)^b^	46	White 91%, Black 4%, Hispanic 3%, Asian 2%, American Indian 0
		10/10					
		S 80		63	56.4 (25–87)^b^	42	White 90%, Black 5%, Hispanic 5%, Asian < 1%, American Indian 0

***Studies***	***Araujo 2010 (30)***	***Settergren 2009 (31,32)***	***Olijhoek 2008 (33)***	***Carcia 2016 (34)***	***Goldberg 2004 (26)***	***Rudofsky 2012 (35)***	***Westerink 2013 (36)***	***Ose 2007 (27,37)***	***Ballantyne 2005 (28)***	***Davidson 2002 (29,38)***
BMI, kg/m^2^	NA	E/S ,29 (27–30)^a^ : S ,28 (26–30)^a^	30.1±2.7	E/S ,36.0±4.4;S ,35.0±4.3	NA	NA	30.0±2.7	E/S ,27.9±4.8 ; S ,28.4±4.9	E/S, 29.8±5.5; A, 30.1±5.6	29±5
BP, mmHg or Hypertension, %	NA	E/S ,150 (150–162)/60 (60–65)^a^ S ,145 (120–160)/60 (57–70))^a^	138±13/89±6	E/S ,38%; S ,31%	NA	E/S ,54.5; S ,70	140±14/85±9	NA	NA	E/S ,30; S ,29
Diabetes or IGT, %	0	E/S ,15; S ,17	NA	E/S ,6.3; S ,0	NA	100	0	NA	NA	E/S ,3; S ,3
CAD, %	NA	100	NA	NA	NA	NA	NA	NA	NA	E/S ,8; S ,6
PAD and Stroke, %	NA	NA	NA	NA	NA	NA	NA	NA	NA	NA
Smoker,%	NA	E/S ,20; S ,17.65	0	E/S ,6.3; S ,6.3	NA	NA	0	NA	NA	E/S ,14; S ,16

Data reported in the form of mean±SD unless indicated.

* mg/dl;

** umol/l;

# u/l;

## mg/dl.

a Data reported in the form of median (quartiles);

b Data reported in the form of mean (range);

Abbreviation: A, atorvastatin; BMI, body mass index; BP, blood pressure; CAD, coronary artery disease; E, ezetimibe; IGT, impaired glucose tolerance; NA, not available; PAD, peripheral arterial disease; RCT, Randomized Controlled Trial; S, simvastatin

### Risk of Bias

**Fig. 2:**
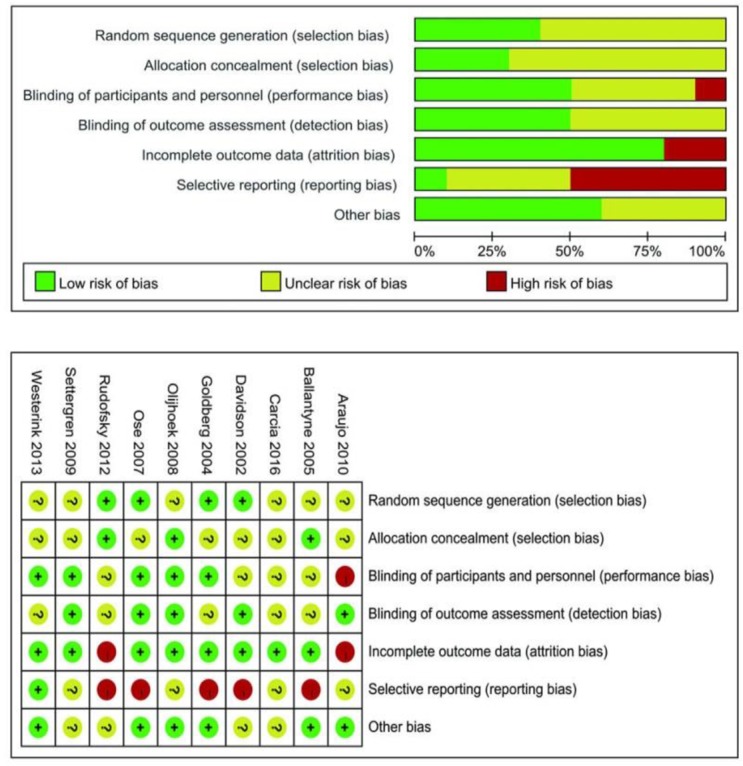
Risk of bias summary

The systematic quality assessment was performed for the included studies using the Cochrane Collaboration Risk of Bias Tool. The overall quality of the included studies was moderate. The methods of random sequence generation were described in four studies (26, 27, 29, 35). Allocation concealment was performed in three studies (28, 33, 35). Random sequence generation and allocation concealment were associated with low selection bias. Only one study was high risk for performance bias (30). Two studies with high risk of bias and eight studies with low risk of bias were key participants in incomplete outcome data (attrition bias) (26–38). However, a half of the included studies were high risk for selective reporting (reporting bias) (26–29, 35). The summary of the risk of bias is shown in Fig. 2.

### Quantitative Data Analysis

#### Effects of ezetimibe/simvastatin treatment and high-dose statin treatment on serum LDL-C

**Fig. 3:**
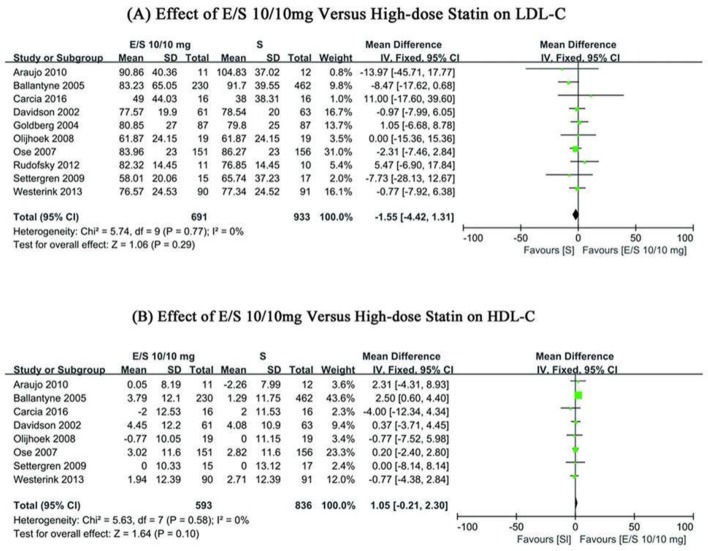
Forest plots showing WMD from baseline in (a) LDL-C, (b) HDL-C;E/S (daily dose of ezetimibe/simvastatin 10/10mg); S, high-dose statin

Overall, 10 comparisons with 691 patients in the ezetimibe/simvastatin group and 933 patients in the high-dose statin group were included in the meta-analysis. There might be low heterogeneity in the LDL-C levels between the included studies (χ^2^=5.74, *I*^2^=0%, *P*=0.77; Fig. 3). The fixed effect model and the pooled WMD were performed with Revman 5.3, and an overall mean LDL-C change from baseline of −1.55 mg/dl (95%CI: −4.42∼1.31, *P*=0.29; Fig. 3) was found in two groups.

#### Effects of ezetimibe/simvastatin treatment and high-dose statin treatment on serum HDL-C

There might be low heterogeneity in the HDL-C levels between the included studies (χ^2^=5.63, *I*^2^=0%, *P*=0.58; Fig. 3).Therefore, the fixed effect model was applied. The pooled WMD of HDL-C was 1.05 (95%CI: −0.21∼2.30, *P*=0.10; Fig. 3) between ezetimibe/simvastatin group and high-dose statin monotherapy group.

#### Effects of ezetimibe/simvastatin and high-dose statin on serum TC and non-HDL-C

**Fig. 4:**
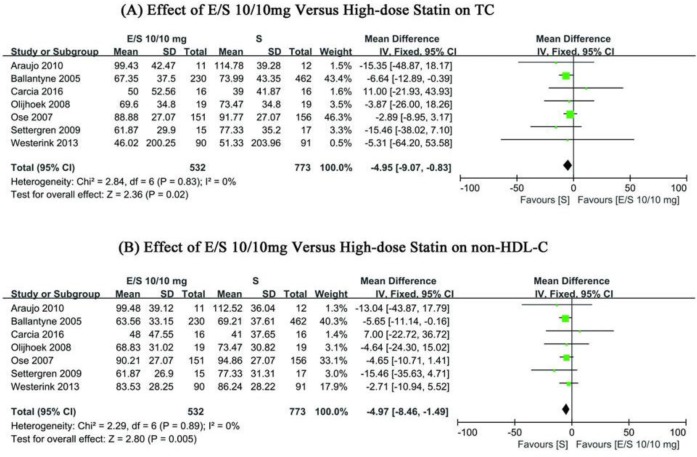
Forest plots showing WMD from baseline in (a) TC, (b) non-HDL-C; E/S (daily dose of ezetimibe/simvastatin 10/10 mg); S, high-dose statin

No significant heterogeneity or low heterogeneity in TC and non-HDL-C were detected for the included studies (χ^2^=2.84, *I*^2^=0%, *P*=0.83, andχ^2^=2.29, *I*^2^=0%, *P*=0.89; Fig. 4). The fixed effect model and pooled WMD were applied with Revman 5.3. In ezetimibe/simvastatin group and high-dose statin group, there was significant difference in TC and non-HDL-C change from baseline of −4.95 mg/dl and −4.97 (95%CI: −9.07∼−0.83, *P*=0.02; 95%CI: −8.46∼−1.49, *P*=0.005; Fig. 4), respectively.

#### Effects of ezetimibe/simvastatin and high-dose statin on serum TG and hs-CRP

**Fig. 5:**
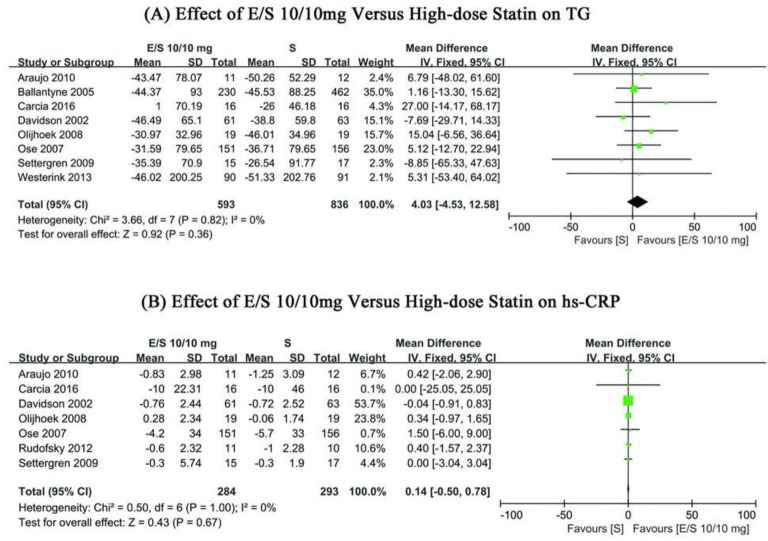
Forest plots showing WMD from baseline in (a) TG, (b) hs-CRP; E/S (daily dose of ezetimibe/simvastatin 10/10mg); S, high-dose statin

The heterogeneity of TG and hs-CRP levels in the included studies was low (χ^2^=3.66, *I*^2^=0%, *P*=0.82, and χ^2^=0.50, *I*^2^=0%, *P*=1.00; Fig. 5). The fixed effect model was used to pool the data. The pooled result suggested that there is close relationship between the ezetimibe/simvastatin group and high-dose statin group, as seen in change from baseline on TG and hs-CRP (WMD: 4.03; 95% CI, −4.53∼12.58; *P*= 0.36; WMD: 0.14; 95% CI, −0.50∼0.78; *P*=0.67; Fig. 5). The sensitive analysis was performed in the pooled result of hs-CRP, because the data from one study (34) presented with significant difference. However, the result of sensitive analysis was not different.

### Publication bias

**Fig. 6:**
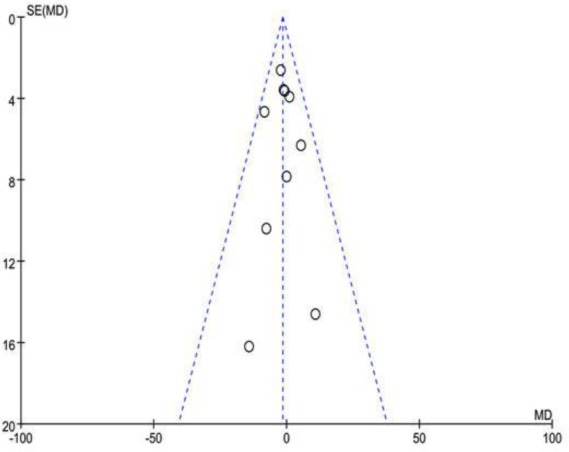
Funnel plot for the evaluation of publication bias. WMD, weighed mean difference; SE, standard error

Assessment of publication bias using the funnel plot by visual inspection was symmetrical (Fig. 6), and it revealed that there is no significant publication bias.

## Discussion

In general, there was an equal overall mean LDL-C, HDL-C, TG, and hs-CRP change from baseline in the ezetimibe plus simvastatin (10 mg/10 mg/d) and high-dose statin groups. However, there was significant difference in TC and non-HDL-C in the two groups, thus showing that the high-dose statin has a more advantageous result. The system review and meta-analysis included ten original studies. The white, Hispanic, black, and Asian races were important participants of these studies, but most of the participants belong to the white, black, and Hispanic races. The basic indices of the original studies, as mentioned before, included BMI, diabetes mellitus or IGT, blood pressure, ischemic stroke, CHD, PAD, liver and renal functions, etc. The sample of terminal outcome in included studies was defined as the sample for system review and meta-analysis. The relationship between the change of LDL-C and hs-CRP from baseline in this meta-analysis and another one (9) was similar in the combined low-dose simvastatin and ezetimibe (ezetimibe/simvastatin 10/10mg) versus high-dose statin groups. Greater LDL-C lowering may be achieved with ezetimibe plus statin as compared with statin. Because, statin was the different dosages and drug species (39).

High-dose statin has many benefits in the treatment of hypercholesterolemia and inflammation, although it has numerous side effects as well. The combination of ezetimibe and low-dose simvastatin contributed to the similar outcome. Similarly, as compared with statin monotherapy, a meta-analysis based on 18 trials of 14,497 patients indicated that the addition of ezetimibe to statin did not increase the risk of increased levels of liver enzymes and CK, myalgia, myopathy, rhabdomyolysis, statin-associated gastrointestinal discomfort, and discontinuations due to AEs (40, 41).

### Limitation of the system review and meta-analysis

The studies included in this system review and meta-analysis had limitations on methodology, particularly the inclusion of a large sample, as seen in some studies (27–29). Hence, the standard grading using GRADE (Grades of Recommendation, Assessment, Development, and Evaluation) resulted in middle rank (Table 2). The systematic review and meta-analysis was performed by searching for studies in English language. Studies not in English were excluded, such as those in Chinese, Russian, German, and Hungarian. Albeit the funnel plots of these non-English studies were symmetrical (Fig. 6), they were still excluded from our meta-analysis. The review had searched a part of the grey literatures, in which was not the formal journal articles. However, due to the presumption of incompleteness of data, the meta-analysis excluded letters, conference summaries/papers, etc. Moreover, the system review and meta-analysis failed to find full papers, particularly, in the grey literature. Thus, some data might be missed.

**Table 2:** The standards grading of GRADE in outcome indicator

***Quality assessment***								
***Outcome indicator***	***Included study***	***Design***	***Quality of methodology***	***Circumstantial evidence***	***Heterogeneity***	***Precision***	***Publication bias***	***Hierarchy of evidence quality***
Ldl-c	10	Rcts	Middle rank^1^	No	High rank	High rank	Middle rank^2^	Middle rank
Hdl-c	8	Rcts	Middle rank^1^	No	High rank	High rank	Middle rank^2^	Middle rank
Tc	7	Rcts	Middle rank^1^	No	High rank	High rank	Middle rank^2^	Middle rank
Tg	8	Rcts	Middle rank^1^	No	High rank	High rank	Middle rank^2^	Middle rank
Non-hdl-c	7	Rcts	Middle rank^1^	No	High rank	High rank	Middle rank^2^	Middle rank
Hs-crp	7	Rcts	Middle rank^1^	No	High rank	High rank	Middle rank^2^	Middle rank

1 Selective reporting (reporting bias);

2 Publication bias; RCTs: Randomized Controlled Trials

In the stage of study selection, the change in LDL-C was necessary. Thus, included studies with other objectives might be decreased, and selective bias should be considered.

The included studies used ezetimibe/simvastatin 10/10 mg, simvastatin 80 mg, and atorvastatin 40/80 mg. The ezetimibe/simvastatin 10/10 mg and simvastatin 80 mg were the primary medications. However, high-dose rosuvastatin was not included. Therefore, the pooled result might be partial to ezetimibe/simvastatin and high-dose simvastatin. Risk of bias was assessed through the Cochrane Collaboration Risk of Bias Tool. The GRADE was used in the quality assessment of the outcome indicator of the included studies. The system review and meta-analysis was performed by different reviewers. The search strategy can be repeated as needed.

## Conclusion

Ezetimibe co-administered with simvastatin (10mg) and high-dose statin monotherapy may provide similar effects in reducing levels of LDL-C, TG, and hs-CRP and in increasing HDL-C. However, the results also suggest that greater TC and non-HDL-C lowering may be achieved with a high-dose statin monotherapy as compared with ezetimibe/simvastatin co-administration. The study determined the efficacy of ezetimibe/simvastatin (10/10 mg) versus high-dose statin. We recommend that more studies should be aimed to determine the efficacy and side effects of ezetimibe co-administered with statin at different dosages and drug species. Moreover, there should be more future studies focusing on the comparison of ezetimibe co-administered with statin versus ezetimibe co-administered with non-statin drugs, particularly PCSK9 inhibitors.

## Ethical considerations

Ethical issues (Including plagiarism, informed consent, misconduct, data fabrication and/or falsification, double publication and/or submission, redundancy, etc.) have been completely observed by the authors.
